# Highly Dispersive Gold Nanoclusters Confined within Micropores of Defective UiO-66 for Highly Efficient Aldehyde Oxidation at Mild Conditions

**DOI:** 10.3390/ijms25126779

**Published:** 2024-06-20

**Authors:** Ming-Qin He, Xin-Yu Chang, Hong-Wei Li, Yuqing Wu

**Affiliations:** 1 State Key Laboratory of Supramolecular Structure and Materials, College of Chemistry, Jilin University, No. 2699 Qianjin Street, Changchun 130012, China; hemq22@mails.jlu.edu.cn (M.-Q.H.); cxy21@mails.jlu.edu.cn (X.-Y.C.); lihongwei@jlu.edu.cn (H.-W.L.); 2 Institute of Theoretical Chemistry, College of Chemistry, Jilin University, No. 2 Liutiao Road, Changchun 130023, China

**Keywords:** defective UiO-66, ultrasmall AuNCs, confined fabrication of AuNCs, furfural esterification, mild condition

## Abstract

The oxidative esterification of aldehydes under mild conditions remains a significant challenge. This study introduces a unique defective UiO-66 to achieve gold nanoclusters (AuNCs) for efficient aldehyde oxidation under mild conditions. The construction and characterization of these materials are thoroughly investigated by techniques of XRD, SEM and TEM images, FT-IR, Raman, and XPS spectrum, emphasizing the unique microporous in defective UiO-66 are conducive to the fabrication of AuNCs. The catalytic performance of the prepared materials in aldehyde oxidation reactions is systematically evaluated, demonstrating the remarkable efficiency of dispersed Au@UiO-66-25 with high-content (9.09 wt%) Au-loading and ultra-small size (~2.7 nm). Moreover, mechanistic insights into the catalytic process under mild conditions (70 °C for 1 h) are provided, elucidating the determination of defective UiO-66 in the confined fabrication of AuNCs and subsequent furfural adsorption, which underlie the principles governing the observed enhancements. This study establishes the groundwork for the synthesis of highly dispersed and catalytically active metal nanoparticles using defective MOFs as a platform, advancing the catalytic esterification reaction of furfural to the next level.

## 1. Introduction

The synthesis of alkyl furoates through the oxidative esterification of furfural (FUR) and fatty alcohols is a crucial method for producing flavor and fragrance components in fine chemicals [1,2]. Significant progress has been achieved in efficiently converting FUR into methyl 2-furoate (MF) through oxidative esterification. For example, Tong’s group utilized prepared Au@UiO-66 for the oxidative esterification of furfural in methanol. Operating at 140 °C for 4 h, they achieved a remarkable 100% conversion of FUR alongside a 100% selectivity towards MF [3]. Similarly, Wang et al. reported the use of surface-cleaned Au_25_@UiO-66-NH_2_, obtained by removing the surface ligand on Au_25_ using the domain-limiting effect of MOF, for the oxidative esterification of furfural. Their results demonstrated that at 100 °C for 4 h, both the conversion of FUR and the selectivity towards MF reached 100% [2]. Nielsen et al. utilized Au/TiO_2_ and CH_3_ONa for the oxidative esterification of furfural at a low temperature; however, achieving a conversion of FUR and a selectivity of MF to 100% required 10–12 h [4]. Despite significant progress in catalyzing furfural oxidative esterification with AuNPs, most reported methods necessitate temperatures higher than 120 °C and reaction times spanning several hours (Table S1) [1,2,3,4,5,6,7,8,9]. Consequently, developing highly efficient AuNPs for the oxidative esterification of furfural under mild conditions, such as at low temperatures and within short reaction times, remains a challenge.

Ultra-small gold nanoclusters (AuNCs) have demonstrated significant potential in various catalytic reactions [10,11]. However, inherently, these small AuNCs tend to aggregate and grow into larger particles. Hence, it is imperative to find a method to restrict gold aggregation, especially under conditions with high gold-loading. In this regard, the strategy of spatial confinement using porous materials such as metal-organic frameworks (MOFs) as support matrices has proven to be effective [12,13]. Specifically, defective UiO-66 not only provides micropores for confining metal loading but also exposes unsaturated Zr(IV) sites due to missing linkers, thereby increasing the availability of acidic sites for reactant adsorption, ultimately enhancing catalysis synergistically [14,15,16].

**Scheme 1:**
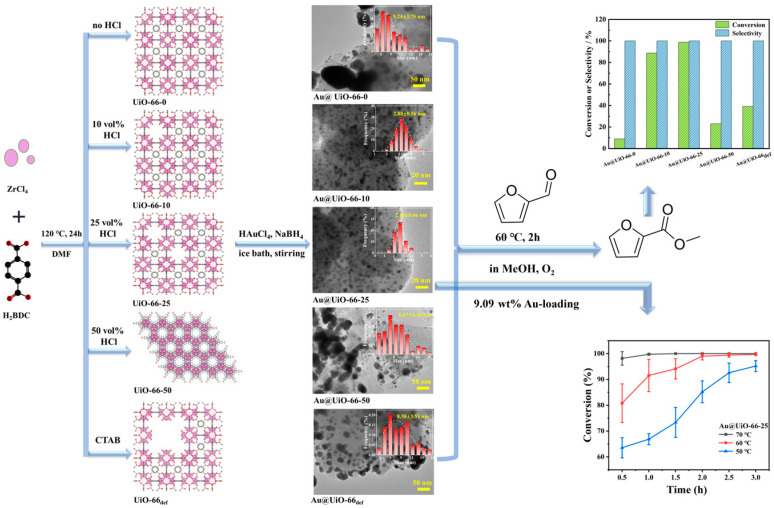
Schematic diagram of the fabrication and catalytic optimization of AuNPs supported by the defective UiO-66 with different levels.

In the present study, therefore, we utilize UiO-66 with varying levels of defects as supports to prepare highly dispersible ultra-small AuNCs (<3.0 nm). Among these, Au@UiO-66-25 achieves full conversion of FUR at 70 °C within one hour (Scheme 1), which can be extended to other aldehydes as well. Thus, by employing hierarchical porous materials hosting high contents of ultrasmall and dispersive gold nanoclusters (AuNCs), this study introduces a novel strategy for the fabrication of highly efficient catalysts under mild conditions.

## 2. Results

### 2.1. Preparation and Characterization of Catalysts

**Figure 1:**
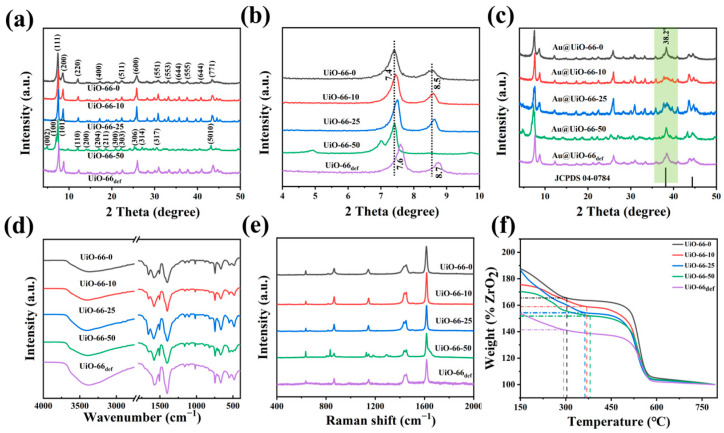
(**a**) XRD patterns of five UiO-66 materials with different defective levels and (**b**) the enlarged part of (**a**), and (**c**) after Au-loading; the corresponding (**d**) FT-IR spectra, (**e**) Raman spectra, and (**f**) TG curves of them, respectively.

Firstly, XRD was used to investigate the crystal structure change of the defective UiO-66 from that of pristine (Figure 1a). Regarding the high similarity between UiO-66-0, UiO-66-10, UiO-66-25 and UiO-66_def_ with that of UiO-66 illustrates all they are crystalline with fcu-topology; however, the XRD pattern of UiO-66-50 is largely different from them in topology, being identified as hcp UiO-66 [17]. Moreover, the enlarged part in Figure 1b shows the obvious moving of two typical diffraction peaks at 7.4° and 8.5° from UiO-66-0 to UiO-66_def_, respectively, indicating a significant defect of UiO-66_def_, while that of others is relatively slight. In addition, Figure 1c exhibits that after Au-loading, the typical diffraction peaks for UiO-66 remain almost unchanged. However, the characteristic peaks at 38.2° for Au(111) [18] are largely different between the five materials. The broader peaks of 38.2° for Au@UiO-66-10 and Au@UiO-66-25 indicate small sizes of AuNPs, which are calculated to be 4.4 and 3.7 nm, respectively, based on Scheller’s formula [19]. In contrast, the sharp and narrow diffraction peaks for Au(111) in Au@UiO-66-0 (19.1 nm), Au@UiO-66-50 (15.1 nm), and Au@UiO-66_def_ (8.4 nm) confirm larger particle sizes of AuNPs in these materials. Therefore, the results indicate that the particle size of the uploaded AuNPs is truly influenced by the defective levels of UiO-66 when using it as a support. Moreover, to investigate the effect of the defects on the chemical bonds of materials in detail, FT-IR and Raman spectra are conducted for each material (Figure 1d,e), where obvious structural differences are illustrated between them. The enlarged FT-IR spectra in Figure S2a indicate the characteristic peak at 550 cm^−1^ being attributed to the vibration of Zr-(μ_3_)-O, suggesting that the Zr(IV) are coordinated to the μ_3_-O bond in terephthalic acid [20]. Notably, the relative intensity of the 550 cm^−1^ peak compared to the 485 cm^−1^ peak (550/485) in UiO-66-25 is significantly weaker than in the four other samples, suggesting more Zr(IV) were exposed in UiO-66-25 and will enhance the adsorption of the substrate as reported [21]. Additionally, in the enlarged Raman spectra (Figure S2b), significant blue shifts at 1143 and 1614 cm^−1^ were observed, which also originated from the defects [22]. Besides, the results of thermogravimetric analysis (TGA) illustrate lower temperatures of framework degradation for all defects than that of UiO-66-0 (Figure 1f), suggesting they are less stable than UiO-66-0. Besides, the results list in the Table S2 visualize the different levels of defects in the five materials. Of note is that the band assignments and detailed descriptions for the FT-IR, Raman spectra, and TGA results are provided in the Supporting Information section.

**Figure 2:**
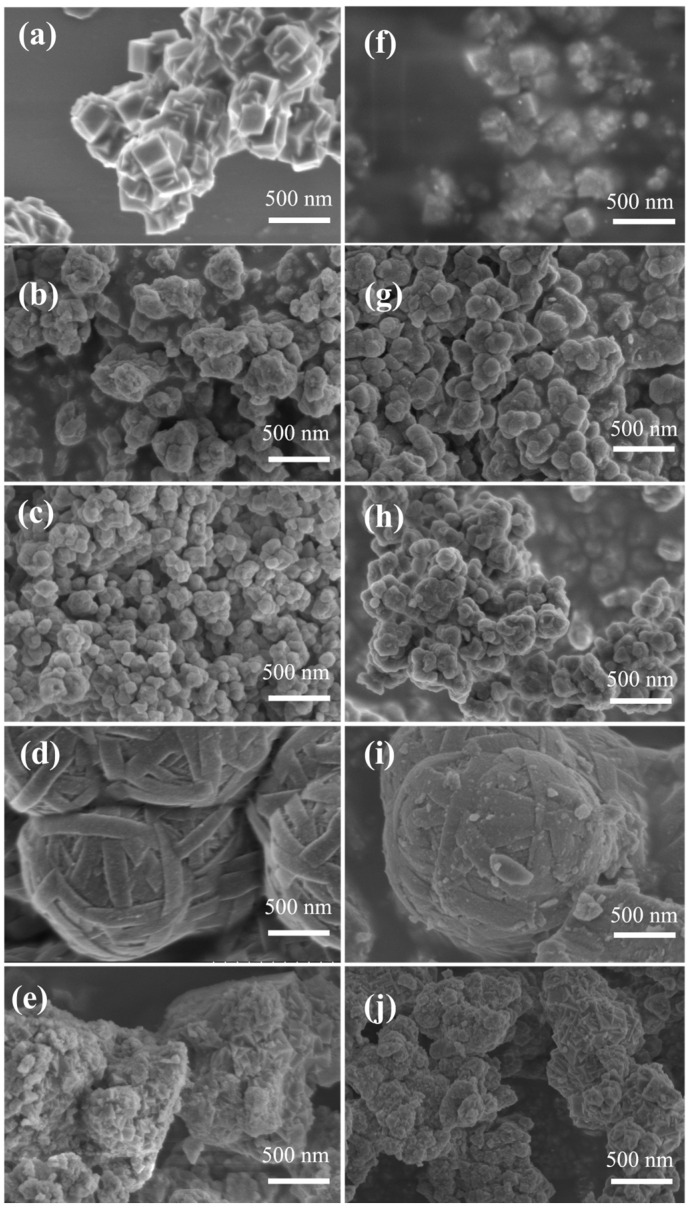
SEM images of (**a**) UiO-66-0 and (**f**) Au@UiO-66-0, (**b**) UiO-66-10 and (**g**) Au@UiO-66-10, (**c**) UiO-66-25 and (**h**) Au@UiO-66-25, (**d**) UiO-66-50 and (**i**) Au@UiO-66-50, and (**e**) UiO-66_def_ and (**j**) Au@UiO-66_def_, respectively.

**Figure 3:**
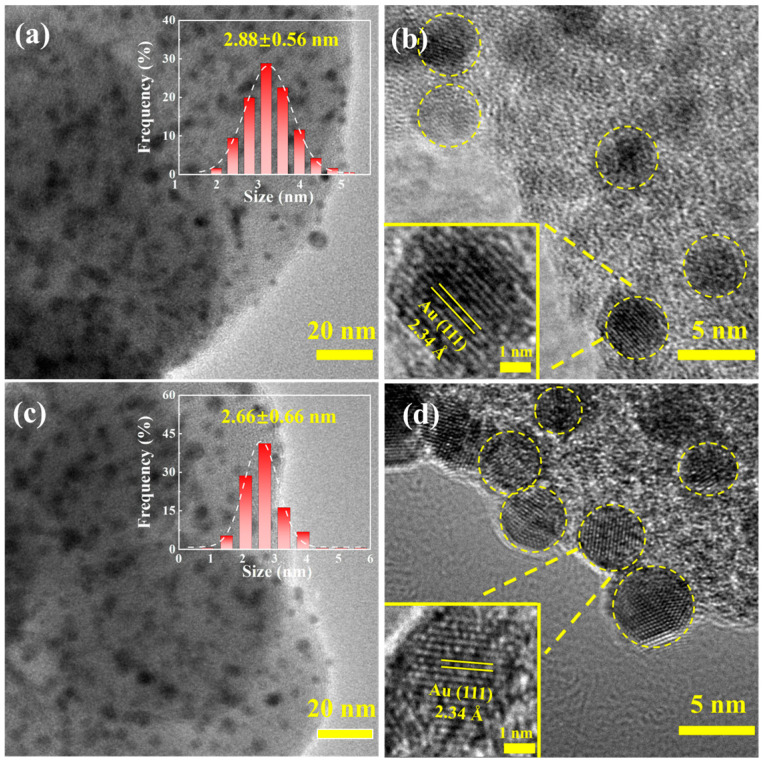
TEM and HRTEM images of (**a**,**b**) Au@UiO-66-10 and (**c**,**d**) Au@UiO-66-25, respectively.

Both the scanning electron microscope (SEM, Figure 2) and high-resolution transmission electron microscopy (HRTEM, Figure 3 and Figure S3) are used to characterize the morphology features of the materials. As illustrated in Figure 2a–e, UiO-66-0 exhibits a cube with a size of ~200 nm, being attributed to the accumulation of octahedral particles [23]. The typical images of UiO-66-10 and UiO-66-25 exhibit heterogeneous aggregates with an atypical cuboctahedron structure, while that of UiO-66-50 becomes a large strip-packed sphere, which is consistent well with XRD results (hcp UiO-66). The results indicate that the acid indeed plays a key role in defecting the crystal of UiO-66 [23]. The representative image of UiO-66_def_ shows large particles composed of aggregates of many small square particles, suggesting that the cluster-missing of UiO-66 induced by CTAB severely disrupts the crystal of MOFs. Besides, the morphologies of Au@UiO-66-X and Au@UiO-66_def_ are also monitored by SEM (Figure 2f,j), which remain almost unchanged after the deposition of AuNCs or AuNPs, suggesting the uploading of gold has little effect on each pristine structure.

As illustrated in Figure 3, the TEM images show that the AuNPs in Au@UiO-66-10 and Au@UiO-66-25 are obviously smaller than those in the other three materials (Figure S2). The statistic on the TEM images revels an average diameter of ~2.9 and ~2.7 nm for Au@UiO-66-10 and Au@UiO-66-25 (Figure 3a,c), respectively, while larger particle size between 8.5–9.3 nm for the other three (Figure S2). Besides, an inter-layer spacing of ~0.23 nm, corresponding to Au(111) [24], is revealed essentially for the Au@UiO-66-10 and Au@UiO-66-25, confirming the deposition of crystalline AuNCs in them. In addition, the mapping of elemental distribution (Figure S3) confirms again the successful loading of AuNCs. Taken together, it indicates that the defective UiO-66 can indeed be used to determine the size of AuNPs. An appropriate structure of defective UiO-66 is especially beneficial for obtaining well-distributed AuNCs with ultrasmall sizes, which lays a foundation for improving the catalytic performance of the composites.

**Figure 4:**
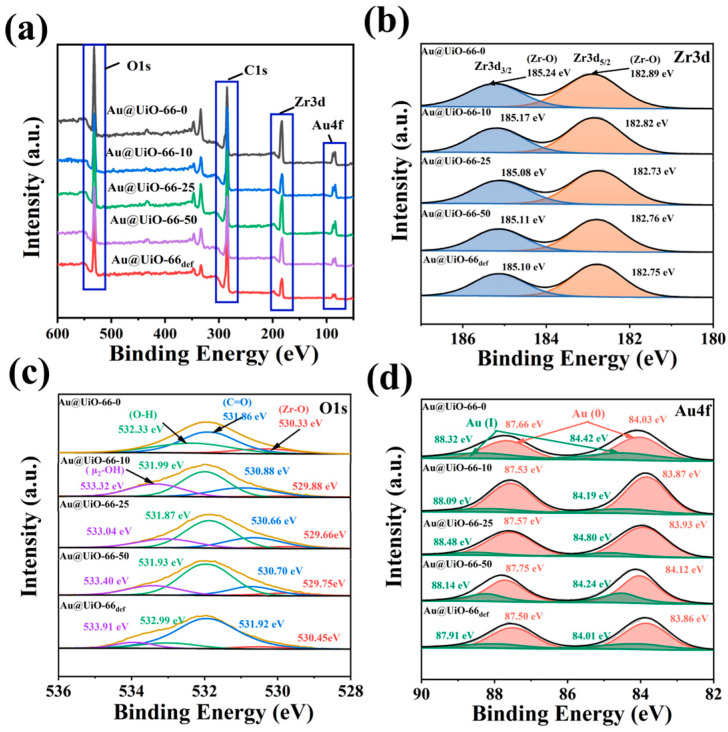
XPS spectra of (**a**) survey, (**b**) Zr3d, (**c**) O1s, and (**d**) Au4f for Au@UiO-66-X and Au@UiO-66_def_, respectively.

The chemical composition and valence state of Au, Zr and O in Au@UiO-66-X and Au@UiO-66_def_ are monitored by employing X-ray photoelectron spectroscopy (XPS; Figure 4a). In the enlarged Zr3d spectra (Figure 4b), the peaks at 185.24 and 182.89 eV are assigned to the binding energies of Zr3d_3/2_ and Zr3d_5/2_, respectively, demonstrating the presence of Zr-O bonds [25]. In the defective UiO-66-supported composites, the binding energy of Zr3d becomes smaller due to the decrease in Zr-O coordination, confirming the generation of defects in UiO-66. In parallel, the O1s spectra of Au@UiO-66-0 show three peaks at 530.33, 531.86, and 532.33 eV (Figure 4c), respectively, is attributed to the oxygen atoms in Zr-O, C=O, and O-H [26,27]. After acidic defection, a new peak at 533.32 eV appears and being recognized as a μ_2_-OH species; meanwhile, the binding energy at 531.86 eV for C=O shifts to a low binding energy upon defection, which is attributed to the unsaturated metal coordination and will be neutralized by Cl^−^. The coordination of Cl^−^ with metal will break the bonds of pristine μ_3_-OH, finally forming μ_2_-OH [28]. In addition, when CTAB was used as a template to construct UiO-66_def_, a new peak at 533.91 eV is shown, being attributed either to the μ_2_-OH.

In addition, the XPS spectra of Au4f (Figure 4d) contain two different peaks at 87.66 and 84.03 eV, being ascribed to the Au4f_7/2_ and Au4f_5/2_. Specifically, the peaks at 88.32 and 84.42 eV are indexed as Au(I) species, while those at 87.66 and 84.03 eV correspond to Au(0), which should be located in the core of each AuNC. Accordingly, the proportions of Au(0) in Au@UiO-66-10 and Au@UiO-66-25 are calculated to be 79.56% and 81.95% in the total of deposited gold, respectively. However, that of Au(0) in the other three materials is obviously lower, being between 64.16 and 71.77%. Of note, although larger AuNPs are involved in the latter three materials, the Au(0) contents there are obviously lower, which are mainly due to the fact that the more stacked AuNPs are shown with an invisible lattice (Figure S1).

### 2.2. Catalytic Assay of Furfural Oxidation by Au@UiO-66-X

First, the catalytic assays of Au@UiO-66-X and Au@UiO-66_def_ for FUR esterification are investigated at 60 °C for 2 h. Considering the electronic states and contributions of AuNPs for catalytic regulation [29], the same amount of Au deposition (6.45 wt%) is kept for each material. As illustrated in Table 1, the conversion rates of Au@UiO-66-0 are quite low (9.04%), while the best one is achieved for Au@UiO-66-25 (98.79%) in following with that of Au@UiO-66-10 (88.71%). Of note, Au@UiO-66-50 and Au@UiO-66_def_ exhibit much lower conversion rates (<40.00%), possibly being attributed to the large particle size of AuNPs in the composites (Figure S1). Besides, the products of FUR oxidation, in the presence of O_2_, could possibly be methyl 2-furoate (MF), 2-(dimethoxymethyl) furan (2-DMF), both at the same time, or others [2,3]. GC-MS results show that only MF is found in all cases (Figure S4), suggesting excellent selectivity of all catalysts for FUR oxidation. Therefore, the following study will focus on the conversion rates of FUR rather than the selectivity. For that, Au@UiO-66-10 and Au@UiO-66-25 will be used as starting materials as more than 88.00% of conversion rates have already been attained for them (Table 1).

Figure S5 shows the time- and temperature-dependent conversion assays of Au@UiO-66-10 and Au@UiO-66-25 for FUR oxidation. First, when the reaction time is fixed at 2 h for Au@UiO-66-10, the conversion rates increase along with the temperature up and achieve 100.0% at 70 °C (Figure S5a). Then, when the temperature is fixed at 70 °C, a 100.0% conversion can be finished in 2 h (Figure S5b). In parallel, the Au@UiO-66-25 shows a similar tendency but better activities than Au@UiO-66-10 (Figure S5c,d). Of note, when the reaction time is extended to 2 h, the complete conversions of FUR are achieved for both. Therefore, at a fixed deposition of 6.45 wt% Au, the best conditions for Au@UiO-66-10 and Au@UiO-66-25 are chosen at 70 °C for 2 h.

Then, the effects of Au-loadings on catalysis are investigated in detail, where the real Au-contents in the composites are measured by inductively coupled plasma emission spectrometry (ICP-OES). To show the gap between composites, the test is started at 50 °C for 2 h (Table S3). However, the results show that FUR cannot be completely oxidized by the two composites at a low temperature of 50 °C in 2 h.

**Figure 5:**
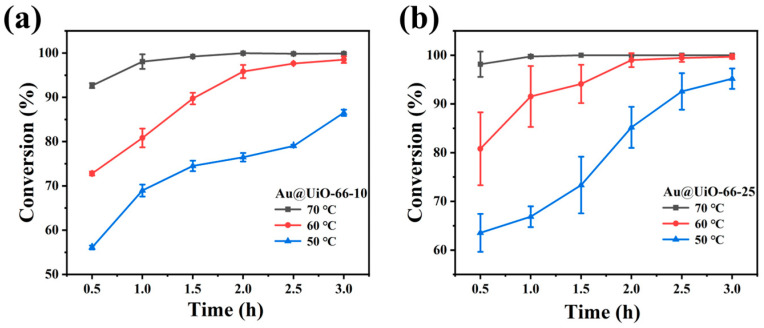
Time-course of conversion rates for (**a**) Au@UIO-66-10 with 8.91 wt% Au-loading, and (**b**) Au@UIO-66-25 with 9.09 wt% Au-loading, on the oxidation of FUR in methanol, at temperature of 50, 60 and 70 °C, respectively. (Reaction conditions: 0.1 mmol FUR, 15 mg catalyst, 2 mg Na_2_CO_3_, in 4 mL methanol, under 0.6 MPa of O_2_).

Next, after the reaction time is extended to 3 h, the Au@UiO-66-25 with Au-loading of 9.09 wt% still cannot achieve a complete oxidative esterification of FUR at 50 °C (96.67%, Figure 5a). However, when the temperature is raised to 60 °C, such Au@UiO-66-25 can achieve a complete esterification of FUR within 2 h, while at 70 °C, the reaction can be completed within 1 h (Figure 5a). In parallel, the Au@UiO-66-10 with 8.91 wt% Au-loading can also achieve a complete FUR conversion at 70 °C, but it needs a longer time (1.5 h; Figure 5b). Therefore, the Au@UiO-66-25 with 9.09 wt% Au-loading exhibits the best conversion efficiency of FUR and a full MF yield in the present study; it can finish within 1 h, at a mild condition as 70 °C (Figure 5a). Noticeably, either Au@UiO-66-10 or Au@UiO-66-25 displays higher catalytic activity than the other three composites (Figure 5), suggesting the crucial role of defective UiO-66 as the support for catalyst construction and will be clarified in next.

Then, a series of cross-coupling reactions between methanol and other four aldehydes are also conducted at 70 °C for 1 h, in the presence of O_2_ (Table S4). A high conversion rate (≥99.40%) is shown for each substrate, which validates the high generality of the developed catalyst for other aldehydes. In addition, the recycling tests on the optimized Au@UiO-66-25 (Figure S6a) further confirm its stability and recoverability in practice as the conversion rates are maintained at no less than 96.25% after four cycle treatments. Besides, the remaining structure of the catalyst examined by XRD (Figure S6b) shows that the microstructure of UiO-66 is still retained well even after four cycles; however, the diffraction peak at 38.2° is getting narrower and sharper. Based on Scherrer’s formula [19], the size of AuNPs is calculated to be 6.3 nm, which suggests that after continuous use, the small particles of AuNCs are fused and grow. Although the reason for that is not yet clear, there is no doubt that larger particles are not conducive to the conversion of FUR. Moreover, the Au@UiO-66-25 prepared a half-year ago was used in the catalytic system at 70 °C for 1 h. Unfortunately, the catalytic efficiency of it has been decreased to 88.62%. As good enough activity has been achieved for the constructed Au@UiO-66-25, further improvements on the stability of AuNCs will be performed in the future.

## 3. Discussion

**Figure 6:**
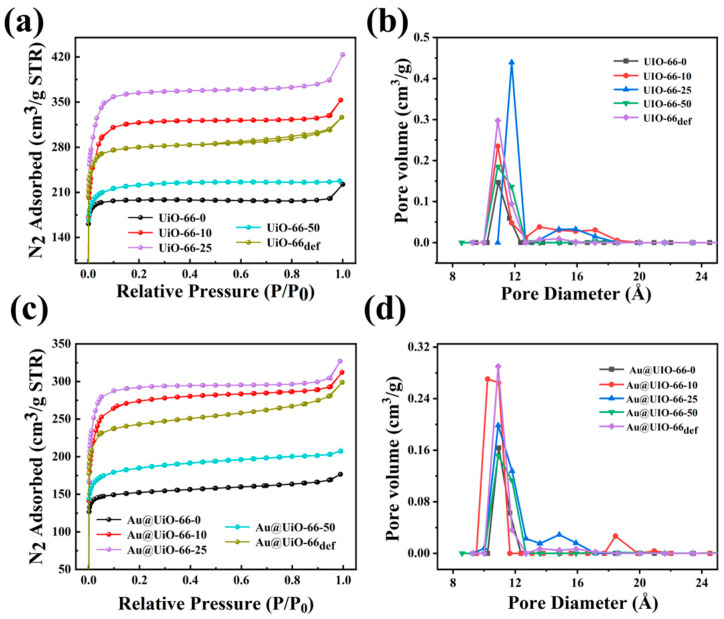
Isotherms of (**a**,**c**) nitrogen adsorption and (**b**,**d**) pore size distributions for UiO-66 and defective UiO-66 based on the DFT model, (**a**,**b**) before and (**c**,**d**) after Au-loading, respectively.

**Figure 7:**
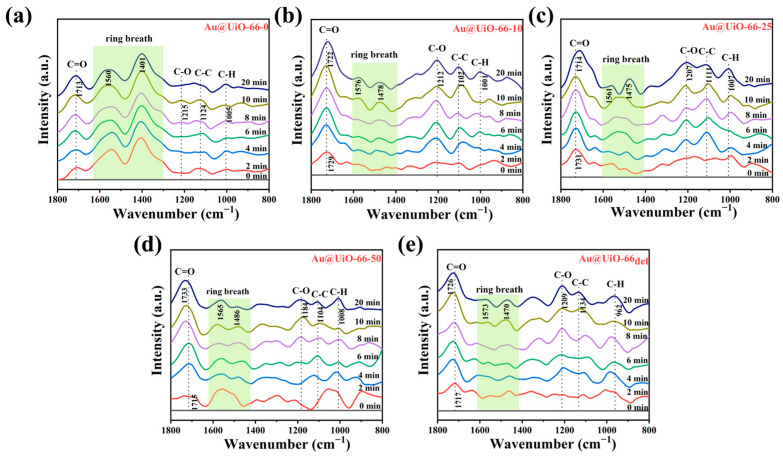
FUR-DRIFTS spectra of (**a**) Au@UiO-66-0, (**b**) Au@UiO-66-10, (**c**) Au@UiO-66-25, (**d**) Au@UiO-66-50, and (**e**) Au@UiO-66_def_, in the range of 1800–800 cm^−1^.

First, the pore size and surface area of defective UiO-66 support AuNPs are investigated by the N_2_ adsorption–desorption isotherms (Figure 6). As illustrated, all the composites exhibit type-1 isotherms, indicating the microporous frameworks [30] of each. Based on the BET measurements, UiO-66-25 has the largest specific surface area of 1092 m^2^/g, followed by UiO-66-10, UiO-66_def_, and UiO-66-0 with a value of 962.7, 844.4 and 671.1 m^2^/g, respectively (Table S5). Of note, they are all larger than that of UiO-66-0 with 593.4 m^2^/g, confirming the successful construction of the defective UiO-66. Besides, the curves of the pore size distribution (Figure 6b,d) demonstrate values of ~1.6 nm for the UiO-66-10 and UiO-66-25, being obviously larger than the others (~1.0 nm). In addition, the UiO-66-10 and UiO-66-25 show larger pore volumes of 0.5463 and 0.6551 cm^3^/g (Table S5), respectively, than the other three, laying foundations for high-content Au-loading. Of note, after gold-loading, the decreased pore volumes confirm the actual AuNC filling in the pores. Meanwhile, the composites show consistent sequence as the pristine defects both for the specific surface areas and pore volumes (Table S5), indicating no disruption of the MOF framework after gold-loading. In conclusion, the results show that the UiO-66-10 and UiO-66-25 process larger specific surface area, pore size and volume, meeting the requirement to upload large amounts of gold. In particular, the large amount of S_micro_ and V_m_**_icros_** in UiO-66-25 contributed to the formation of small and dispersive AuNCs (~2.7 nm, Figure 3). Therefore, it is the defective UiO-66 that intrinsically determines AuNC uploading, where the size matching and spatial confinement contributed essentially to the Au hosting. Besides, as indicated by the FUR-DRIFTS spectrum performed (Figure 7), the large specific surface area and pore volume of UiO-66-25 can improve the absorbability of furfural at the surface, which further improves the catalytic performance of Au@UiO-66-25 on the FUR esterification.

As displayed in Figure 7, the peak that appeared at ~1720 cm^−1^ is ascribed to the stretching model of C=O (ν_C=O_) [31], and those at ~1400 cm^−1^ and ~1550 cm^−1^ are assigned to the vibrations of C=C and furan ring breath in FUR, respectively [31,32,33]. Besides, the peaks between 1000–1215 cm^−1^ are assigned to the C-O, C-C stretching, and the C-H bending within the furan ring [31,34]. Typically, when C=O is adsorbed at the surface, the linear orientation of it results in a red shift of ν_C=O_, while the lying bridge absorption leads to the blue shift of it [35]. As illustrated, the peak positions of ν_C=O_ are located at 1729 and 1731 cm^−1^ initially for Au@UiO-66-10 and Au@UiO-66-25, but they shift to 1722 and 1714 cm^−1^, respectively, with the incubation time. However, non- or blue shifts of ν_C=O_ are shown for another three materials in parallel, suggesting lying bridge absorption of C=O at the surface. The clear red shifts of ν_C=O_ in Au@UiO-66-10 and Au@UiO-66-25 indicate the linear adsorption of C=O at the catalyst surface, which weakens the density of electronic cloud around C=O and results in a lower wavenumber [31]. In this case, the C=O is easily activated upon the binding with the exposed Zr(IV) with stronger Lewis acidities [36,37,38]. Therefore, the experiment of NH_3_-temperature programmed desorption (NH_3_-TPD) will be performed to clarify the quantity and intensity of surface acidity in different catalysts.

As depicted in Figure S7, the desorption peak areas of UiO-66-10 and UiO-66-25 for NH_3_ are the most prominent among the test groups, indicating a higher number of acidic sites after acidification. However, there is only a slight difference between UiO-66-50 and UiO-66-0. Combined with the intensity ratio of 550/485 in the FT-IR spectra (Figure 1d and Figure S2a), this suggests that the number of acidic sites in UiO-66-50 is not greater than that in UiO-66-0. Table 2 summarizes the quantities of acid sites derived from the NH_3_-TPD curves for each variant, with UiO-66-25 exhibiting the highest quantified value. Both UiO-66-25 (1.468 mmol/g) and UiO-66-10 (1.353 mmol/g) possess a higher concentration of acid sites, leading to a more efficient conversion rate of FUR compared to the other three materials. After the capping ligands were removed, the exposed Zr(IV) can be regarded as Lewis acid sites [15]. As indicated in the FT-IR spectra (Figure S2a), the intensity ratio of 550/485 in UiO-66-10, UiO-66-25, and UiO-66_def_ is lower compared to UiO-66-0 and UiO-66-50, indicating that more defective sites were produced in the first three samples. Moreover, Table 2 shows that the number of acid sites in UiO-66_def_ is lower than in UiO-66-10, which is attributed to the introduction of HCl in UiO-66-10. Notably, UiO-66-25 possesses the highest number of defects and acidic sites, contributing to the enhanced adsorption of reactants on the catalyst surface [39,40]. Hence, it is the levels of acid-induced defects in UiO-66-X that determine the number of acid sites in the materials, consequently enhancing catalytic activity.

Hence, Au@UiO-66-25, with an actual Au-loading of 9.09 wt% and smaller, more dispersed size (~2.7 nm), exhibits the most superior catalytic activity in enhancing FUR conversion to MF, achieving completion within just 1 h at 70 °C. This exceptional catalytic performance can be primarily attributed to several factors. Firstly, the substantial BET-specific surface area and pore volume of UiO-66-25 enhance the deposition of a large quantity of AuNCs, providing a solid foundation for the provision of abundant gold sites for catalytic activity. Secondly, the abundance of micropores within UiO-66-25 facilitates the confined assembly of ultrasmall and well-dispersed AuNCs inside, resulting in the creation of more active gold sites and thus excellent catalytic performance. Consequently, the structural disparities between UiO-66-25 and other variants confer upon it a specific configuration with a higher density of active sites for Au deposition. Notably, the substantial exposure of Zr(IV) within UiO-66-25 functions as Lewis acid sites, effectively adsorbing and activating the C=O group in furfural in a linear fashion, thereby significantly enhancing catalytic performance.

## 4. Materials and Methods

### 4.1. Materials

Zirconium chloride (ZrCl_4_) was purchased from Macklin Reagent (Shanghai, China) (purity ≥ 99%), terephthalic acid (TPA), chromatographic grade methanol and tetrachloroauric acid trihydrate (HAuCl_4_·3H_2_O) were purchased from Aladdin (Shanghai, China) (purities ≥ 99%). Methanol (AR) and sodium borohydride (NaBH_4_; purity ≥ 96%) were purchased from Sinopharm Chemical Reagent (Shanghai, China). Hydrochloric acid (AR) was purchased from Xilong Chemical Reagent (Guangzhou, China). N, N-dimethylformamide (DMF) (AR) was purchased from Tianjin Tiantai Chemical Company Limited (Tianjin, China). Besides, cetyltrimethylammonium bromide (CTAB) was purchased from Sigma-Aldrich (Shanghai, China) (purity ≥ 99%). Sodium carbonate (Na_2_CO_3_) was purchased from Tianjin Guangfu Science and Technology Development Company Limited (Tianjin, China) (purity ≥ 99%).

### 4.2. Synthesis of Defective UiO-66

The synthesis procedure of defective UiO-66 with linker missing follows a previous report utilizing HCl as a modulator [17]. For that, 0.57 mmol solid ZrCl_4_ was dissolved in 9 mL DMF and mixed with varying volumes of concentrated HCl from 0 to 9 mL, respectively. This mixture was ultrasonicated in a water bath at room temperature (RT) until all ZrCl_4_ was completely solubilized. Next, 0.57 mmol of terephthalic acid was added to the mixture, and the total volume was adjusted to 18 mL with DMF. After further sonification for 30 min, the mixture was transferred to a pressure-resistant bottle and heated at 120 °C for 24 h to ensure complete reaction between the feed materials. Upon cooling to RT, the precipitate was filtered and washed three times with pure DMF (5 mL), followed by methanol (5 mL) three times. The products were then filtered until all residual solvents were removed. The final products of UiO-66 without or with different levels of linker missing were termed as UiO-66-X, where X stands for the volume percentage of the concentrated HCl in the reaction system, i.e., 0, 10, 25, or 50 vol%, respectively.

The defective UiO-66 with Zr(IV) center missing was named UiO-66_def_, which was synthesized using a surfactant-assisted method [41]. For that, 103.8 mg CTAB was introduced into the solution of ZrCl_4_ in DMF, prepared as described previously without the addition of HCl. Subsequently, the remaining procedures mirrored those outlined for the synthesis of UiO-66-0. Following vacuum filtration to remove residual solvent, the product was dissolved in 150 mL methanol solution containing 1.5 mL concentrated HCl, which was refluxed at 80 °C for 24 h to remove excessive CTAB [42]. The precipitate was then filtered and washed twice with 0.4 mM × 5 mL ammonia solution, followed by pure methanol twice (5 mL).

### 4.3. Synthesis of Au@UiO-66 Materials

Au@UiO-66 was synthesized using a solvent impregnation–reduction method [43]. In the typical experiment, 50 mg defective UiO-66-X was dissolved in 6 mL methanol/water (1:1 *v*/*v*) solvents under sonication. Subsequently, 1.75 mL × 10 mM HAuCl_4_ was added, and the solution was stirred in an ice bath for one hour. Then, 2.8 mL × 25 mM NaBH_4_ solution was added to the mixture drop-wise and stirred in an ice bath for 3 h. Finally, the precipitate was centrifuged and vacuum-dried at 90 °C.

### 4.4. Equipment for Characterizations

Thermogravimetric analysis was performed using a TGA550 (Waters Corporation, Milford, MA, USA), while infrared spectra were measured using a VERTEX 80V (Bruker, Berlin, Germany). Raman spectra were recorded using a T64000 (HORIBA JOBIN YVON, Palaiseau, France) and X-ray diffraction (XRD) was conducted with a Rigaku D/max2550 (Rigaku, Tokyo, Japan) diffractometer using Cu-Kα radiation with a scanning speed of 3° per minute between two thetas of 4° and 50°. Besides, scanning electron microscopy (SEM) measurements were performed using a Hitachi Regulus 8100 (Hitachi, Tokyo, Japan) and transmission electron microscopy (TEM) measurements were taken using a JEM-2200FS (Jeol Ltd., Tokyo, Japan). The gold content of all samples was quantified by inductively coupled plasma emission spectrometry (ICP-OES, Agilent 725, Frederick, CO, USA). Nitrogen adsorption/desorption isotherms were recorded on specific surface area with an iPore 400 pore (PhysiChem Instruments Ltd., Beijing, China) size analyzer. Specific surface area values were calculated based on the Bruner–Emmett–Taylor (BET) model [44]. Prior to measurement, the samples were degassed under a dynamic vacuum at 200 °C for 4 h. X-ray photoelectron spectroscopy (XPS) measurements were performed on a Thermo Scientific ESCALAB 250Xi (Thermo Fisher Scientific, Waltham, MA, USA) with monochromatized Al target. The FUR-DRIFTS spectra were carried out on a VERTEXV 80 V FT-IR spectrometer (Bruker, Germany) equipped with a liquid nitrogen-cooled MCT detector. NH_3_-TPD (Temperature-Programmed Desorption) was performed on AutoChem II 2920 V5.02 (Micromeritics, Norcross, GA, USA).

### 4.5. Oxidative Esterification Assay of Furfural in MeOH

The catalytic reaction occurs within a 50 mL high-pressure reactor equipped with magnetic stirring and an automatic temperature control system. Typically, 15 mg catalyst, 0.1 mmol furfural, 2 mg Na_2_CO_3_ and 4 mL MeOH were mixed, followed by exchanging air with pure O_2_ three times. Once pressurized with O_2_ (0.6 MPa) in the high-pressure reactor, the mixture is heated to the desired temperature (ranging from 50 to 90 °C) and maintained at that temperature for varying durations (from 0.5 to 3 h) under stirring. Upon completion of the reaction, the solution in the reactor is cooled in an ice bath and then centrifuged to separate the catalyst from the solution. Subsequently, the conversion of furfural (FUR) and selectivity to methyl-2-furoate (MF) are determined based on the results obtained from gas chromatography-mass spectrometry (GC-MS), calculated using the following equations, respectively:

$$\mathrm{FUR}\;\mathrm{conversion}\;\left(\%\right)=\frac{\left(\mathrm{Initial}\;\mathrm{FUR}\;\mathrm{amount}/\mathrm{mol})-(\mathrm{Final}\;\mathrm{FUR}\;\mathrm{amount}/\mathrm{mol}\right)}{\mathrm{Initial}\;\mathrm{FUR}\;\mathrm{amount}/\mathrm{mol}}\times 100\%\;\mathrm{MF}\;\mathrm{yield}\;\left(\%\right)=\frac{\mathrm{Final}\;\mathrm{MF}\;\mathrm{amount}/\mathrm{mol}}{\mathrm{Initial}\;\mathrm{FUR}\;\mathrm{amount}/\mathrm{mol}}\times 100\%\;\mathrm{MF}\;\mathrm{yield}\;\left(\%\right)=\frac{\mathrm{Final}\;\mathrm{MF}\;\mathrm{amount}/\mathrm{mol}}{\mathrm{Initial}\;\mathrm{FUR}\;\mathrm{amount}/\mathrm{mol}}\times 100\%$$

## 5. Conclusions

In this investigation, we synthesized a series of gold nanoclusters (AuNCs) confined within the micropores of defective UiO-66. Among these, Au@UiO-66-25, with a 9.09 wt% Au-loading, exhibited remarkable catalytic activity, achieving complete conversion of furfural to methyl-2-furoate (MF) under mild conditions at 70 °C within 1 h. Detailed mechanistic studies revealed the determination of defective UiO-66 in enabling the confined fabrication of ultrasmall, well-dispersed AuNCs, and the subsequent furfural adsorption, both synergistically effects culminated in the exceptional catalytic performance of Au@UiO-66-25. Furthermore, the recycling tests on Au@UiO-66-25 maintained high furfural conversion even after four cycles, demonstrating its good practicality, although the stability of AuNCs needs to be improved in the future. Particularly noteworthy was its ability to facilitate the full cross-coupling of methanol with various other aldehydes, underscoring its versatility for aldehyde oxidation. Consequently, the conceptual framework established in this study offers fresh insights into the critical factors governing MOF-based catalyst design, specifically for efficient aldehyde oxidation.

## Figures and Tables

**Table 1:** Oxidative transformation of furfural with different catalysts Au@UiO-66-X or Au@UiO-66_def_ in methanol, respectively ^1^.

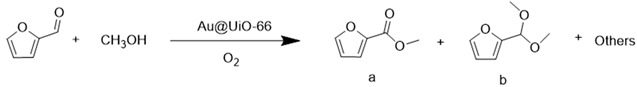				
No.	Catalysts	Con. (%)	Sel. (%, a)	Sel. (%, b)
1	Au@UiO-66-0	9.04	100.0	N.D.
2	Au@UiO-66-10	88.71	100.0	N.D.
3	Au@UiO-66-25	98.79	100.0	N.D.
4	Au@UiO-66-50	23.07	100.0	N.D.
5	Au@UiO-66_def_	39.14	100.0	N.D.

^1^ Reaction conditions: furfural (0.1 mmol), catalyst (15 mg, ~6.45 wt% Au incorporation) and Na_2_CO_3_ (2 mg) in 4 mL methanol, in the presence of 0.6 MPa O_2_, at 60 °C for 2 h. N.D.—not detectable.

**Table 2:** Acid quantity derived from the NH_3_-TPD curve of different materials.

Samples	UiO-66-0	UiO-66-10	UiO-66-25	UiO-66-50	UiO-66_def_
Acid quantity (mmol/g)	1.039	1.353	1.468	1.014	1.247

## Data Availability

Data are contained within the article.

## Supplementary Materials

The following supporting information can be downloaded at: https://www.mdpi.com/article/10.3390/ijms25126779/s1. References [1,2,3,4,5,6,7,8,9,17,23,30,41,45,46,47,48,49,50,51] are cited in the Supplementary Materials.
