# Supplementary material for: Highly Dispersive Gold Nanoclusters Confined within Micropores of Defective UiO-66 for Highly Efficient Aldehyde Oxidation at Mild Conditions
Source: Int J Mol Sci. 2024 Jun 20;25(12):6779. doi: 10.3390/ijms25126779 (PMC11203797; doi:10.3390/ijms25126779)
Supplement: Supplementary file 1 [file ijms-25-06779-s001.zip › ijms-3001941-supplementary.pdf]

Highly Dispersive Gold-nanoclusters Confined within Mi-croporous of Defective UiO-66 for High-efficient Aldehyde Oxidation at Mild Conditions

(ESI)

Ming-Qin He,[a,b] Xin-Yu Chang,[a,b] Hong-Wei Li,[a,b] and Yuqing Wu[a,b*]

[a] State Key Laboratory of Supramolecular Structure and Materials, College of Chemistry, Jilin University, No. 2699 Qianjin Street, Changchun 130012, P. R. China.

[b] Institute of Theoretical Chemistry, College of Chemistry, Jilin University, No. 2 Liutiao Road, Changchun 130023, P. R. China.

* Correspondence: wuyuqing@jlu.edu.cn.

1. The defects effects on the chemical bonds of defective UiO-66

In Fig. 1d, five materials differ at peak positions of 480 cm^{-1}, 750 cm^{-1}, and 1663 cm^{-1}, respectively. The bands around 480 cm^{-1} belong to the μ_3-OH stretch [1–3]. The appearances of them confirm the formation of Zr_6 clusters, as well as the coordination between Zr(IV) and the carboxyl groups of BDC in all materials. Besides, the decrease of band at 480 cm^{-1} in the spectrum of UiO-66-50 indicates a loss of local symmetry in the Zr cluster, being similar to the distortion observed for the dehydroxylation of Zr_6 cluster due to the removal of bridging OH groups [4]. In addition, the peak at 745 cm^{-1} is assigned to the stretching model of Zr-O-Zr in UiO-66 [5]. There is a certain difference in the intensity ratio between it and the adjacent band at 660 cm^{-1}, among the five materials. Meanwhile, the band at 1663 cm^{-1} is ascribed to the -C=O stretching of the BDC linker, the intensity decrease (or even disappearance) of it in UiO-66_{def} suggests that the C=O bond in the UiO-66 framework was disrupted severely by the introduction of CTAB during the synthesis, which may result in a cluster loss [6,7]. Furthermore, the peak at 1663 cm^{-1} for the -C=O is broadened and suppressed in UiO-66-50, suggesting excessive volume of HCl

possibly disrupts it in the framework. In summary, FT-IR spectroscopy indicates that there are obvious structural differences among the five defective materials. In particular, UiO-66-10 and UiO-66-25 have certain defects, yet are still structurally similar to UiO-66-0.

The Raman spectra of UiO-66-X, and UiO-66_{def} are illustrated in Fig. 1e. UiO-66-0 revealed characteristic peaks at 635, 866, 1146, 1450 and 1617 cm^{-1}, being assigned to the in-plane bending of aromatic ring, symmetric stretching of C=C/COO (in-phase), breathing and in-plane bending of aromatic ring, stretching of C−C, and the asymmetric stretching of O-C-O [8]. As enlarged in Fig. S2b that the peaks near 1146 and 1617 cm^{-1} are somewhat blue-shifted with defect levels of the materials. The blue-shift in the Raman band implies that such vibrational models require higher energies in the defective UiO-66 than in the non-defect one. In addition, the peak near 1450 cm^{-1} is broad in UiO-66-0 than in other defects (Fig. S2b). It is evident that because of the altered conformation of UiO-66-50, the peaks of it exhibit different stretching vibrations near 850, 1140, 1295, and 1617 cm^{-1}, compared to the other materials (Fig. 1e). Therefore, the structural differences exist between the five materials are revealed either well by the Raman spectrum.

2. The defects effects on the thermostability of defective UiO-66

In Fig. 1f, the weight loss occurred in the temperature range of 150-380 °C is attributed to the dehydration of Zr cluster, while the final weight loss above 380 °C can be attributed to the lose either of the linker or Zr center. Besides, in detail, TGA analysis can also be used to estimate the numbers of missing linker in the defective UiO-66 crystal [9]. The results list in the Table S2 visualize the different levels of defects in the five materials. It can be seen that both the UIO-66-X and UiO-66_{def} exhibited 2.06-2.90 BDC linkers per Zr_6 node in the respective framework. The high BDC linker deficiency in the UIO-66_{def} framework (3.94) is further understood by the magnitude of the weight loss trend in UIO-66_{def}, which was lower than the UiO-66-0. Meanwhile, there is a more pronounced difference in the structure stability of the five materials (Fig. 1f). For example, the temperature for the release of hydrated water can be last until 380 °C for the defective crystal of UiO-66-50. However, the temperature

for structural degradation of the framework for all defects are lower than that of UiO-66-0, indicating the less stable of them than the UiO-66-0 [10].

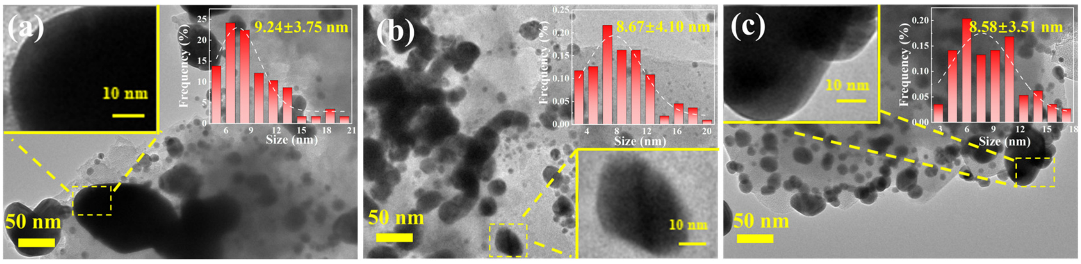

Fig. S1. Typical TEM images of (a) Au@UiO-66-0, (b) Au@UiO-66-50 and (c) Au@UiO-66_{def}, respectively.

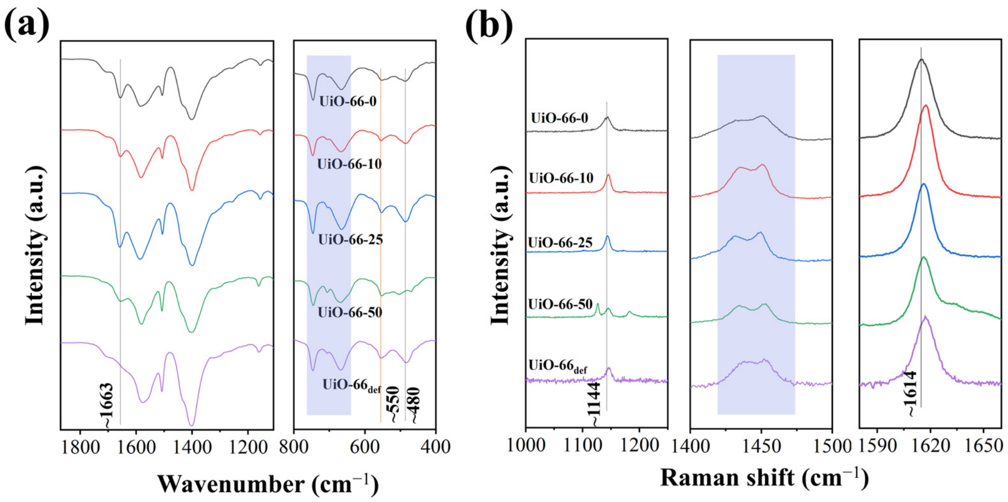

Fig. S2. Localized magnification of FT-IR (a) and Raman spectra (b) at different ranges.

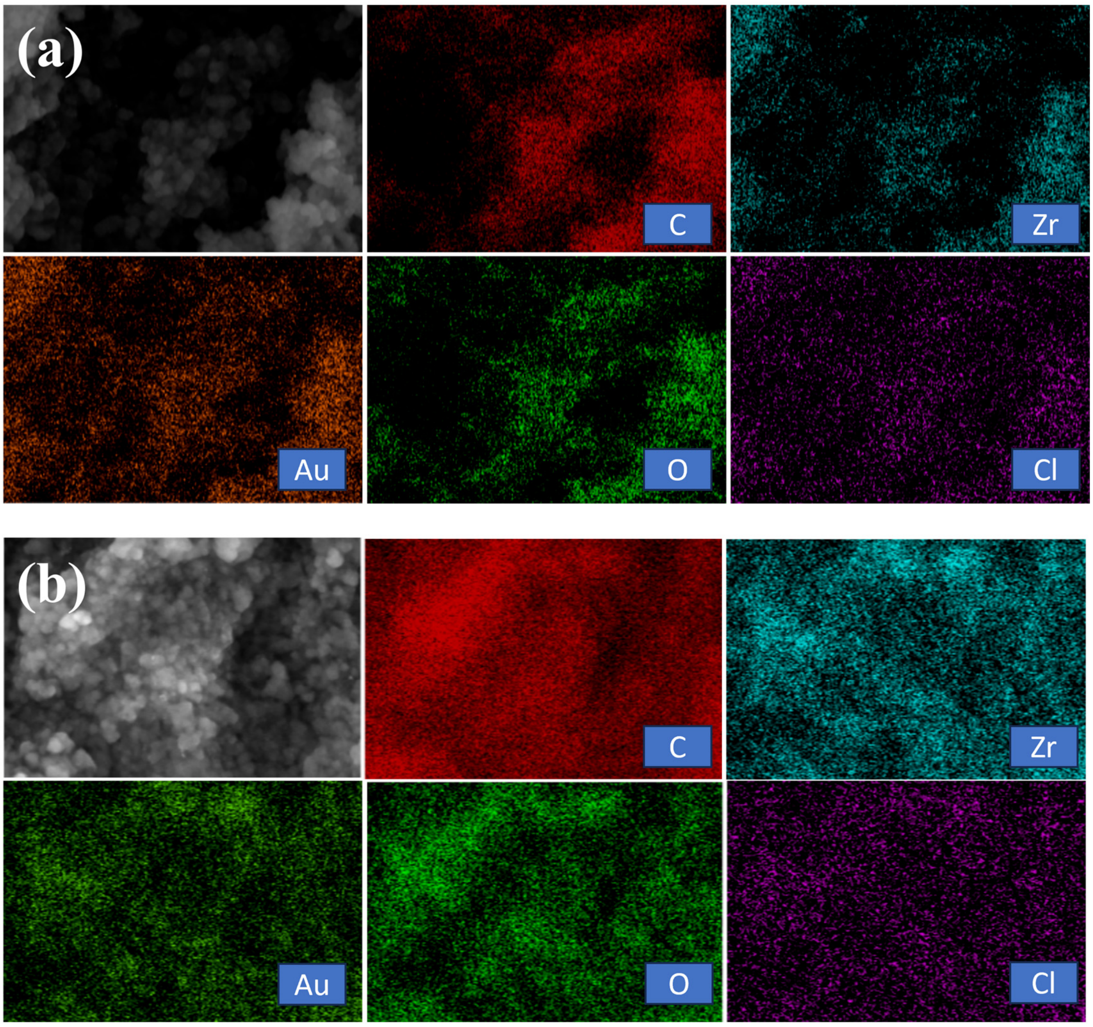

Fig. S3. EDS mapping of elements in Au@UiO-66-10 (a) and Au@UiO-66-25 (b), respectively.

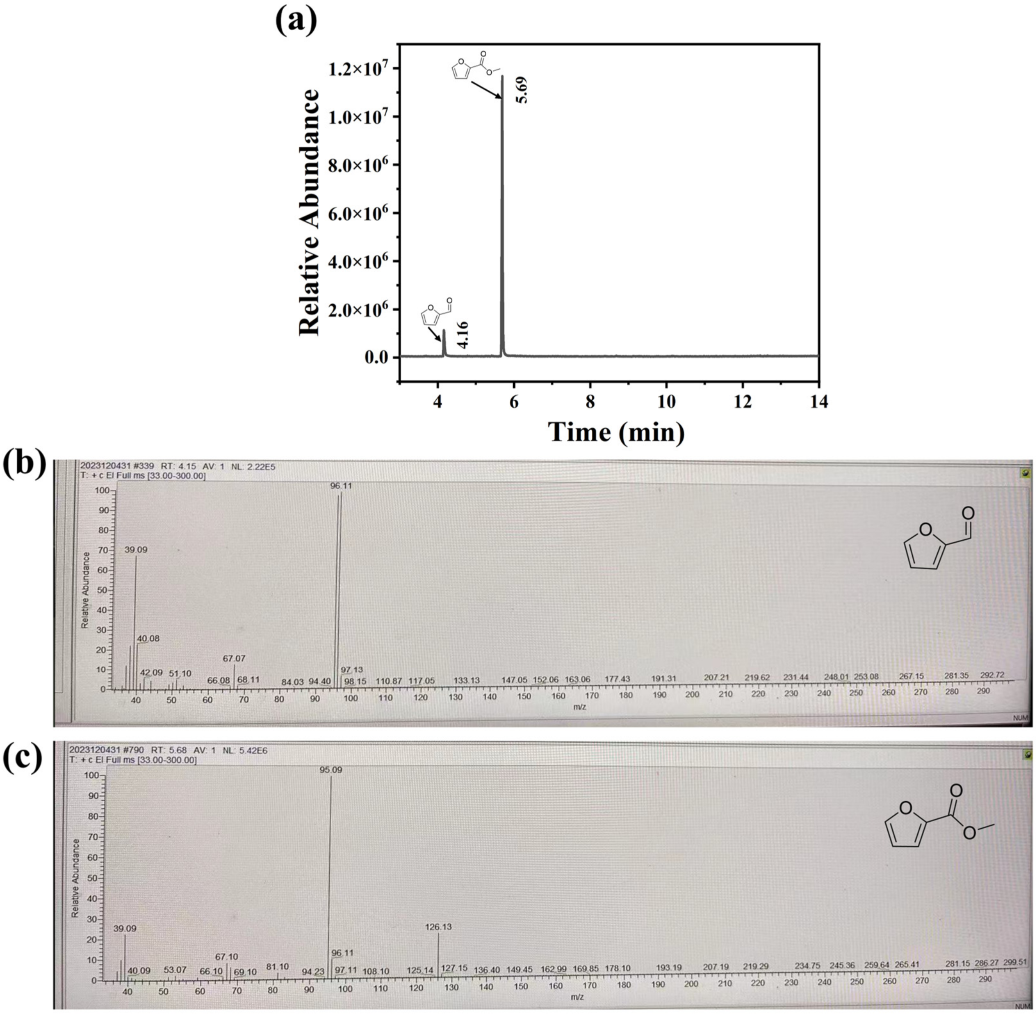

Fig. S4. Gas chromatogram of furfural catalyzed oxidation to ester at Au-deposition of 8.59 wt% Au@UiO-66-10 (a) and mass spectra of furfural (b) and methyl furoate (c). (Reaction conditions: 0.1 mmol FUR, 15 mg catalyst, 2 mg Na_2CO_3, in 4 mL methanol, under 0.6 Mpa of O_2, at 60 °C for 1.5 h).

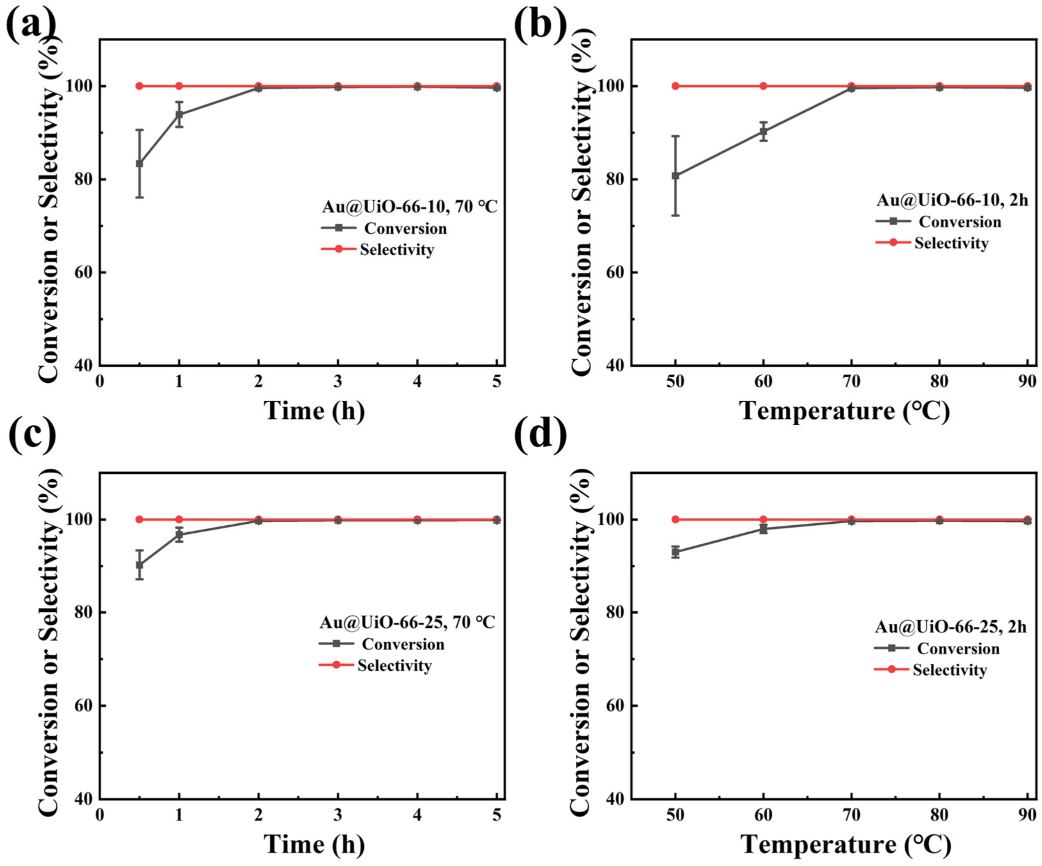

Fig. S5. The (a, c) time- and (b, d) temperature-dependent conversion rates of Au@UiO-66-10 or Au@UiO-66-25 (with an Au deposition of 6.45 wt%) for furfural esterification, respectively (Reaction conditions: 0.1 mmol FUR, 15 mg catalyst, 2 mg Na_2CO_3, in 4 mL methanol, under 0.6 MPa of O_2).

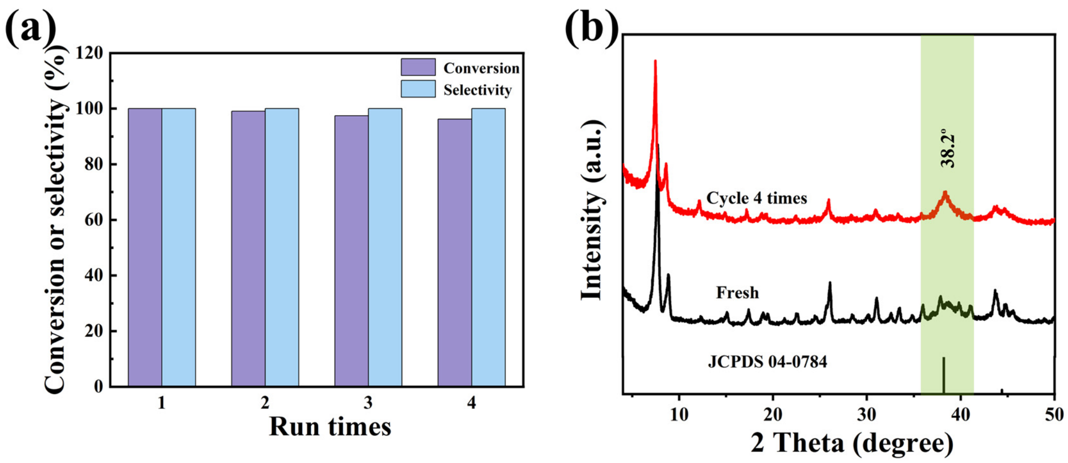

Fig. S6. (a) The recycling experimental results and (b) XRD of Au-loading of 9.09 wt% Au@UiO-66-25 in the oxidative esterification of FUR with methanol. (Reaction conditions: 0.1 mmol FUR, 15 mg catalyst, 2 mg Na_2CO_3, in 4 mL methanol, under 0.6 Mpa of O_2, at 70 °C for 1 h).

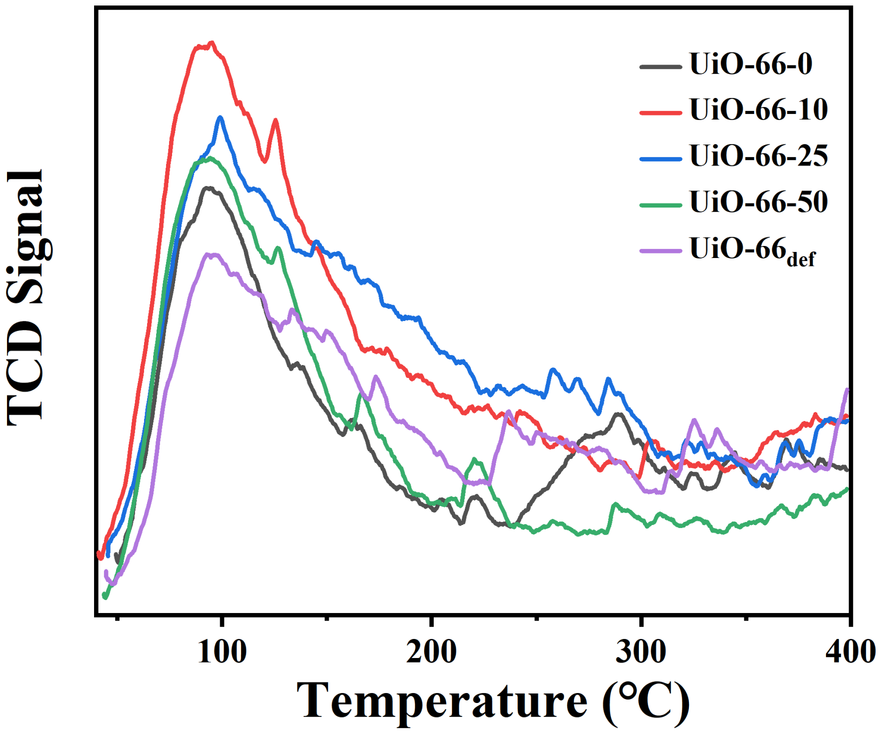

Fig. S7. NH_3-TPD profiles of UiO-66-X and UiO-66$_{def}$ at different temperatures.

Table S1. Comparison of catalytic performances of the reported catalysts to the present study, for the conversion of FUR to MF in methanol.

Ref	Condition	Catalyst	Furfural Conversion (%)	MF Selectivity (%)
[11]	0. 3 MPa O_2 ,140 °C, 4h	Au@UiO-66 (I-H)<br>Au@UiO-66 (I-S)	100.0<br>62.10	100.0<br>100.0
[12]	0. 6 MPa O_2, 100.0 °C, 4 h	Au_{25}/UIO-66<br>Au_{25}/ZrO_2<br>Au_{25}@UIO-66<br>Au_{25}@UIO-66-NH_2	68.60<br>57.90<br>99.90<br>100.0	100.0<br>100.0<br>100.0<br>100.0
[13]	22 °C, 0.1 MPa O_2 (bubbling conditions), 10–12 h	Au/TiO_2+ CH_3ONa	~100.0	~100.0
[14]	120 °C, 0.6 MPa O_2, 3 h	AZ150<br>AZ300<br>AZ500<br>AZ600<br>AZ650	99.00<br>98.00<br>100.0<br>57.00<br>24.00	94.00<br>94.00<br>98.00<br>76.00<br>60.00
[15]	120 °C, 0.6 MPa O_2, 90 min	Au/ZrO_2<br>Au/CeO_2<br>Au/TiO_2	82.00<br>66.00<br>20.00	92.00<br>~70.00<br>~90.00
[16]	120 °C, 0.6 MPa O_2, 90 min	$Ce_{90}Au_{300}$<br>$Ce_{110}Au_{300}$<br>$Ce_{300}Au_{300}$<br>$Ce_{90}Au_{500}$<br>$Ce_{110}Au_{500}$<br>$Ce_{500}Au_{500}$	29.00<br>29.00<br>54.00<br>6.000<br>28.00<br>74.00	100.0<br>100.0<br>100.0<br>100.0<br>100.0<br>100.0
[17]	140 °C, 0.3 MPa O_2, 4 h	Au/FH + K_2CO_3	93.00	99.00
[18]	120 °C, 1.5 MPa O_2, 3 h	5%Au/CMK-3	99.70	99.60
[19]	140 °C, 1.0 MPa O_2, 1h	Au/Al_2O_3	99.80	100.0
[20]	120 °C, 2.0 MPa O_2, 3h	Cu/Co_2P@C-NS-36 nm	99.00	99.00
This work	70 °C, 0.6 MPa O_2, 1.5 h<br>70 °C, 0.6 MPa O_2, 1 h	Au@UIO-66-10<br>Au@UIO-66-25	>99.00<br>100.0	100.0<br>100.0

Table S2. Number of BDC linkers, missing linkers, formula, and molar mass of per Zr_6 node in UiO-66-X and UiO-66_{def}, which are evaluated based on the TGA analysis*.

Samples	Nos of BDC linkers (6 – x)	Nos of missing linkers (x)	Formula	Molar mass
UiO-66-0	3.290	2.710	$Zr_6O_{8.71}(BDC)_{3.29}$	1227
UiO-66-10	2.900	3.100	$Zr_6O_{9.10}(BDC)_{2.90}$	1169
UiO-66-25	2.650	3.350	$Zr_6O_{9.35}(BDC)_{2.65}$	1132
UiO-66-50	2.600	3.400	$Zr_6O_{9.40}(BDC)_{2.60}$	1124
UiO-66_{def}	2.060	3.940	$Zr_6O_{9.94}(BDC)_{2.06}$	1044

*TGA analysis of each parameter in different materials was performed using previously reported methods [9].

Table S3. The effects of Au-loading on FUR esterification when using UiO-66-10 and UiO-66-25 as carrier for catalyst construction*, respectively.

Catalysts	Au Load (wt%)		Conversion of FUR (%)	Selectivity for MF (%)
	Theoretical	ICP-OES		
Au@UiO-66-10	2.30	2.41	9.780	100.0
	4.49	4.00	57.50	100.0
	6.45	5.42	68.31	100.0
	8.59	8.91	77.14	100.0
	10.9	9.12	51.22	100.0
Au@UiO-66-25	2.30	2.94	21.92	100.0
	4.49	4.28	43.79	100.0
	6.45	4.98	67.93	100.0
	8.59	8.27	82.58	100.0
	10. 9	9.09	88.19	100.0

*Reaction conditions: FUR (0.1 mmol), catalyst (15 mg), Na_2CO_3 (2 mg), in methanol (4 mL) with O_2 (0.6 MPa), being reacted at 50 °C for 2 h.

Table S4. Oxidative esterification of various furfuryl derivatives with methanol over 10.00 wt% Au@UiO-66-25*

Entry	Substrate	Product	Conversion (%)	Selectivity (%)
1	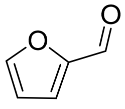	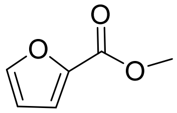	100.0.0	100.0.0
2	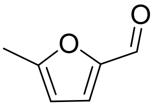	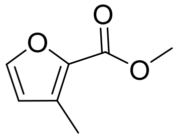	99.50	100.0.0
3	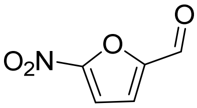	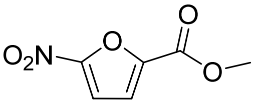	100.0.0	100.0.0
4	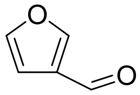	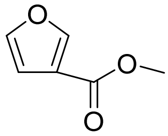	99.40	100.0.0
5	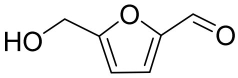	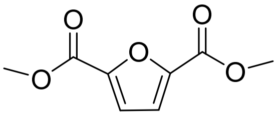	99.60	70.00

*Reaction conditions: 0.1 mmol substrate, 15 mg catalyst, 2 mg Na_2CO_3, in 4 mL methanol, under 0.6 MPa of O_2, 70 °C, 1h.

Table S5. Structural properties of UiO-66-X, UiO-66_{def}, Au@UiO-66-X, and Au@UiO-66_{def} used in the present study.

**Samples**	**S_{BET} (m^2/g)**	**S_{micro} (m^2/g)**	**S_{ext} (m^2/g)**	**V_{Total} (cm^3/g)**	**V_{micro} (cm^3/g)**	**$D_{Average}$ (nm)**
UiO-66-0	593.4	48.44	545.0	0.3440	0.2586	1.160
Au@UiO-66-0	461.1	53.96	407.1	0.2731	0.1951	1.180
UiO-66-10	962.7	144.7	818.0	0.5463	0.3481	1.130
Au@UiO-66-10	837.1	132.3	704.8	0.4826	0.3012	1.150
UiO-66-25	1092	155.5	936.9	0.6551	0.4151	1.200
Au@UiO-66-25	882.5	124.6	758.0	0.5059	0.3322	1.150
UiO-66-50	671.1	63.64	607.4	0.3520	0.2808	1.050
Au@UiO-66-50	565.2	70.57	494.6	0.3208	0.2309	1.140
UiO-66_{def}	844.4	106.2	738.2	0.5047	0.3430	1.200
Au@UiO-66_{def}	743.1	115.9	627.1	0.4623	0.2877	1.240

References

[1] X. Chen, Y. Li, Q. Fu, H. Qin, J. Lv, K. Yang, Q. Zhang, H. Zhang, M. Wang, An efficient modulated synthesis of zirconium metal–organic framework UiO-66, RSC Advances 12 (2022) 6083–6092. https://doi.org/10.1039/D1RA07848H.

[2] E. Geravand, F. Farzaneh, M. Ghiasi, Metalation and DFT studies of metal organic frameworks UiO-66(Zr) with vanadium chloride as allyl alcohol epoxidation catalyst, J. Mol. Struct. 1198 (2019) 126940. https://doi.org/10.1016/j.molstruc.2019.126940.

[3] Y. Han, M. Liu, K. Li, Y. Zuo, Y. Wei, S. Xu, G. Zhang, C. Song, Z. Zhang, X. Guo, Facile synthesis of morphology and size-controlled zirconium metal–organic framework UiO-66: the role of hydrofluoric acid in crystallization, CrystEngComm 17 (2015) 6434–6440. https://doi.org/10.1039/C5CE00729A.

[4] C.A. Clark, K.N. Heck, C.D. Powell, M.S. Wong, Highly Defective UiO-66 Materials for the Adsorptive Removal of Perfluorooctanesulfonate, ACS Sustain. Chem. Eng. 7 (2019) 6619–6628. https://doi.org/10.1021/acssuschemeng.8b05572.

[5] M. Fayyazi, A.R. Solaimany Nazar, M. Farhadian, S. Tangestaninejad, Adsorptive removal of ibuprofen to binary and amine-functionalized UiO-66 in the aquatic environment: synergistic/antagonistic evaluation, Environ. Sci. Pollut. Res. 29 (2022) 69502–69516. https://doi.org/10.1007/s11356-022-20703-2.

[6] X. Zhang, Y. Yang, L. Song, J. Chen, Y. Yang, Y. Wang, Enhanced adsorption performance of gaseous toluene on defective UiO-66 metal organic framework: Equilibrium and kinetic studies, J. Hazard. Mater. 365 (2019) 597–605. https://doi.org/10.1016/j.jhazmat.2018.11.049.

[7] X. Shi, X. Zhang, F. Bi, Z. Zheng, L. Sheng, J. Xu, Z. Wang, Y. Yang, Effective toluene adsorption over defective UiO-66-NH_2: An experimental and computational exploration, J. Mol. Struct. 316 (2020) 113812. https://doi.org/10.1016/j.molliq.2020.113812.

[8] P.H.M. Andrade, N. Henry, C. Volkringer, T. Loiseau, H. Vezin, M. Hureau, A. Moissette, Iodine Uptake by Zr-/Hf-Based UiO-66 Materials: The Influence of Metal Substitution on Iodine Evolution, ACS Appl. Mater. Interfaces 14 (2022) 29916–29933. https://doi.org/10.1021/acsami.2c07288.

[9] A.K. Kar, R. Sarkar, A.K. Manal, R. Kumar, S. Chakraborty, R. Ahuja, R. Srivastava, Unveiling and understanding the remarkable enhancement in the catalytic activity by the defect creation in UIO-66 during the catalytic transfer hydrodeoxygenation of vanillin with isopropanol, Appl. Catal. B Environ. 325 (2023) 122385. https://doi.org/10.1016/j.apcatb.2023.122385.

[10] R. Wei, C.A. Gaggioli, G. Li, T. Islamoglu, Z. Zhang, P. Yu, O.K. Farha, C.J. Cramer, L. Gagliardi, D. Yang, B.C. Gates, Tuning the Properties of Zr6O8 Nodes in the Metal Organic Framework UiO-66 by Selection of Node-Bound Ligands and Linkers, Chem. Mater. 31 (2019) 1655–1663. https://doi.org/10.1021/acs.chemmater.8b05037.

[11] L. Ning, S. Liao, X. Liu, P. Guo, Z. Zhang, H. Zhang, X. Tong, A regulatable oxidative valorization of furfural with aliphatic alcohols catalyzed by functionalized metal-organic frameworks-supported Au nanoparticles, J. Catal. 364 (2018) 1–13. https://doi.org/10.1016/j.jcat.2018.04.030.

[12] H. Wang, X. Liu, W. Yang, G. Mao, Z. Meng, Z. Wu, H.-L. Jiang, Surface-Clean Au_{25} Nanoclusters in Modulated Microenvironment Enabled by Metal–Organic Frameworks

for Enhanced Catalysis, J. Am. Chem. Soc. 144 (2022) 22008–22017. https://doi.org/10.1021/jacs.2c09136.

[13] I.S. Nielsen, E. Taarning, K. Egeblad, R. Madsen, C.H. Christensen, Direct aerobic oxidation of primary alcohols to methyl esters catalyzed by a heterogeneous gold catalyst, Catal. Lett. 116 (2007) 35–40. https://doi.org/10.1007/s10562-007-9086-9.

[14] M. Signoretto, F. Menegazzo, L. Contessotto, F. Pinna, M. Manzoli, F. Boccuzzi, Au/ZrO_2: an efficient and reusable catalyst for the oxidative esterification of renewable furfural, Appl. Catal. B Environ. 129 (2013) 287–293. https://doi.org/10.1016/j.apcatb.2012.09.035.

[15] F. Menegazzo, M. Signoretto, F. Pinna, M. Manzoli, V. Aina, G. Cerrato, F. Boccuzzi, Oxidative esterification of renewable furfural on gold-based catalysts: Which is the best support?, J. Catal. 309 (2014) 241–247. https://doi.org/10.1016/j.jcat.2013.10.005.

[16] M. Manzoli, F. Menegazzo, M. Signoretto, G. Cruciani, F. Pinna, Effects of synthetic parameters on the catalytic performance of Au/CeO_2 for furfural oxidative esterification, J. Catal. 330 (2015) 465–473. https://doi.org/10.1016/j.jcat.2015.07.030.

[17] X. Tong, Z. Liu, L. Yu, Y. Li, A tunable process: catalytic transformation of renewable furfural with aliphatic alcohols in the presence of molecular oxygen, Chem. Commun. 51 (2015) 3674–3677. https://doi.org/10.1039/C4CC09562F.

[18] R. Radhakrishnan, S. Thiripuranthagan, A. Devarajan, S. Kumaravel, E. Erusappan, K. Kannan, Oxidative esterification of furfural by Au nanoparticles supported CMK-3 mesoporous catalysts, Appl. Catal. Gen. 545 (2017) 33–43. https://doi.org/10.1016/j.apcata.2017.07.031.

[19] J. Tian, X. Cheng, G. Liu, Z. Ren, Y. Wang, T. Wei, D. Zhang, Y. Guo, Efficient aerobic oxidative esterification of furfural to methylfuroate over Au/Al_2O_3 catalysts in base-free medium, New J. Chem. 48 (2024) 7651–7659. https://doi.org/10.1039/D3NJ05231A.

[20] Y. Dong, G. Chen, X. Zuo, J. Li, J. Yu, G. Zhang, J. Kuang, I. Akpinar, L. Peng, X. Tang, J.-C. Dong, L. Lin, P. Lyu, S. Yang, J.-F. Li, Nitrogen, Sulfur Co-doped Hollow Carbon-Encapsulated Cu/Co_2 P for Selective Oxidation Esterification of Furfurals, ACS Catal. 14 (2024) 6565–6576. https://doi.org/10.1021/acscatal.4c01035.
